# Experimental and Computational Approach to Studying Supramolecular Structures in Propanol and Its Halogen Derivatives

**DOI:** 10.1021/acs.jpcb.3c02092

**Published:** 2023-10-17

**Authors:** Kinga Łucak, Anna Z. Szeremeta, Roman Wrzalik, Joanna Grelska, Karolina Jurkiewicz, Natalia Soszka, Barbara Hachuła, Daniel Kramarczyk, Katarzyna Grzybowska, Beibei Yao, Kamil Kamiński, Sebastian Pawlus

**Affiliations:** † Institute of Physics, Faculty of Science and Technology, University of Silesia in Katowice, 75 Pułku Piechoty 1, 41-500 Chorzów, Poland; ‡ Institute of Chemistry, Faculty of Science and Technology, University of Silesia in Katowice, Szkolna 9, 40-006 Katowice, Poland

## Abstract

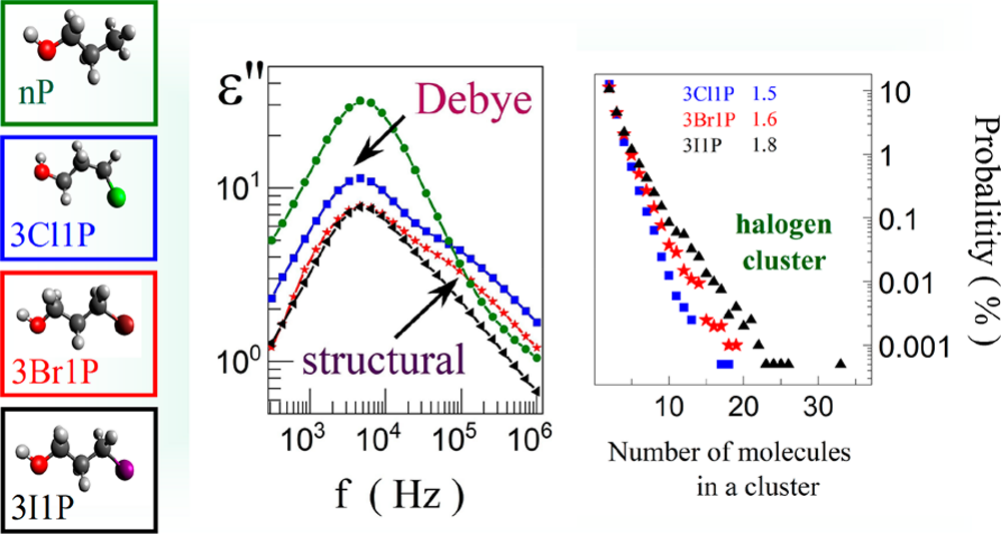

A series of four alcohols, *n*-propanol and its halogen (Cl, Br, and I) derivatives, were selected to study the effects of variation in polarity and halogen-driven interactions on the hydrogen bonding pattern and supramolecular structure by means of experimental and theoretical methods. It was demonstrated on both grounds that the average strength of H-bonds remains the same but dissociation enthalpy, the size of molecular nanoassemblies, as well as long-range correlations between dipoles vary with the molecular weight of halogen atom. Further molecular dynamics simulations indicated that it is connected to the variation in the molecular order introduced by specific halogen-based hydrogen bonds and halogen–halogen interactions. Our results also provided important experimental evidence supporting the assumption of the transient chain model on the molecular origin of the structural process in self-assembling alcohols.

## 1 Introduction

Monohydroxy alcohols (MAs), due to their unique physical and chemical properties and close similarity to water, are at the center of attention to many research groups, and industry oriented on the cryogenic applications or development of new solvents.^1−3^ One of their most fascinating features is an exponential response function–called Debye (D) relaxation, which is a source of hot debate. It is commonly observed in dielectric measurements of aliphatic MAs, irrespective of the position of hydroxyl units in the molecule.^4−6^ According to the current knowledge, the dynamic properties of the D process for associating liquids are an emanation of their complex internal structure driven by the formation of various supramolecular clusters through hydrogen bonds (HBs). However, the exact molecular origin of this characteristic polarization decay is still yet to be addressed.^5,7−18^ Herein one can briefly mention that there are several possible explanations for the nature of this process.^19−25^ Among them, the transient chain model (TCM) proposed by Gainaru and co-workers^26^ provides the most commonly accepted description of the molecular origin of the D relaxation. The model postulates that the D mode appears due to changes in the dipole moment associated with the attachment and detachment of molecules to the ends of the H-bonded chains.

To find a deeper connection between the dynamical properties of the D process and the architecture or size of the supramolecular associates, authors focused their attention on the investigations of the two basic classes of monohydroxy alcohols.^8,9,17,18^ The first one obeys alcohols differing in the position of the OH group in the carbon skeleton or in the chain length of the backbone with a constant location of the OH group. These chemical modifications led to different patterns of association. For example, chain-like motifs of HBs are preferred in the primary alcohols where OH group is located at the end of the molecular backbone (e.g., propanol, 2-ethyl-1-hexanol),^13^ while in other cases, ring-type or more branched assemblies of HBs, at the expense of chain structures, are preferred.^25^ Importantly, variation in the architecture of the supramolecular clusters is reflected in the change of amplitude, relaxation time, and time scale separation from the structural process, characterizing the Debye mode.

The second category of alcohols includes molecules in which the attached functional group is devoid of the dipole moment. For example, a phenyl group added to the alkyl chain behaves as a steric hindrance, preventing the formation of effective HBs. It leads to the inhibition of the self-association phenomenon and the unification of the time scales of the α- and D processes. As a consequence of that, a single relaxation process in dielectric loss spectra is detected.^27−30^

Surprisingly, much less is done on the systems where an additional dipole moment comparable to the one generated by the hydroxyl moiety is introduced into the structure. The most prominent and simplest examples of such systems are halogen derivatives of MAs, which constitute a new class of MAs. One can expect that introducing an atom of a high electronegativity to the molecule causes additional dipole–dipole halogen–halogen interactions as well as halogen-based HBs that may compete with the “classical” HBs. As a consequence, the association process may be disturbed. Therefore, it is so important to investigate the impact of additional polar units on the size and architecture of the supramolecular structures, local molecular order, driving forces leading to the structuring, etc.

**Figure 1:**
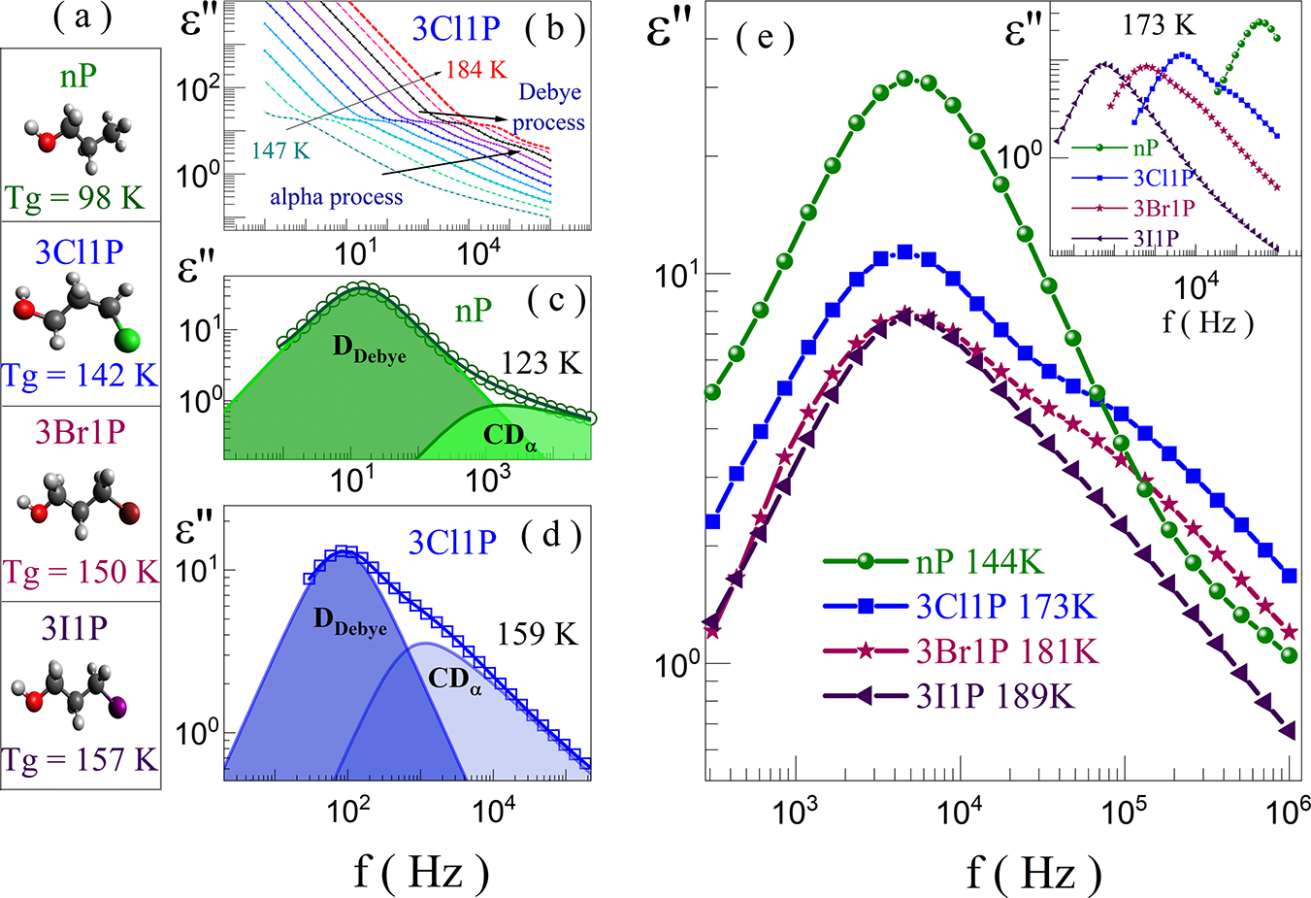
Molecular structure models and glass transition temperatures of the studied alcohols: nP, 3Cl1P, 3Br1P, and 3I1P (a). Dielectric loss spectra for 3Cl1P (b). Dielectric loss spectrum for: nP at 123 K (c) and 3Cl1P (d) at 159 K obtained after subtraction of dc-conductivity. In (c) and (d), the respective solid lines are the results of fitting with the use of the Debye and Cole–Davidson functions (open circles, nP; and open squares, 3Cl1P) show the experiential data. Comparison of the D process characterized by the same relaxation times for all of the studied alcohols (e). The inset shows the dielectric loss spectra after the subtraction of conductivity.

Herein, we chose to study “ordinary alcohol”, *n*-propanol (nP), and its halogen derivatives: 3-chloro-1-propanol (3Cl1P), 3-bromo-1-propanol (3Br1P), and 3-iodo-1-propanol (3I1P). Their structures are shown in Figure 1a. Broadband dielectric (BDS) and Fourier transform infrared (FTIR) spectroscopy, differential scanning calorimetry (DSC) supported by the molecular dynamics simulations (MDS), and density functional theory (DFT) calculations were applied to investigate the competition between the additional dipole–dipole halogen–halogen, halogen-based H-bonds, and the classical H-bonds. Consequently, we could construct a thorough picture of the impact of these interactions on the association nature of the halogen alcohols (XAs, where X indicates the halogen atom: Cl, Br, or I).

## 2 Experimental Section

### 2.1 Broadband Dielectric Spectroscopy (BDS)

The dielectric studies were performed by means of a Novocontrol BDS spectrometer equipped with an Alpha Impedance Analyzer and a Quatro Cryosystem. The capacitor used for the dielectric measurements consisted of two parallel plates of 10 mm diameter made of stainless steel, distanced with two glass fibers of 100 μm thickness and sealed with a Teflon ring. The dielectric spectra were collected in the frequency range of 10^–1^–10^6^ Hz at a quasi-static conditions, that is after stabilization of the temperature for 3 min prior to each measurements using nitrogen gas with a precision better than 0.2 K. The temperature-dependent measurements were performed with a step of Δ*T* = 2 K for 3Cl1P, 3Br1P, and 3I1P and nP with the step of Δ*T* = 3 K.

### 2.2 Fourier Transform Infrared Spectroscopy (FTIR)

FTIR experiments were performed by using a Thermo Scientific Nicolet iS50 spectrometer. The spectra were recorded with a resolution of 4 cm^–1^ as an average of 16 scans. The frequency region covered the range of 400–4000 cm^–1^. Low-temperature FTIR measurements (from 299 to 143 K) were carried out using a Linkam THMS 600 heating/cooling stage (Linkam Scientific Instruments Ltd., Surrey, UK). The spectra were collected every 2 K with the cooling rate of 2 K min^–1^. The halogenated alcohols (HAs) were placed between CaF_2_ windows, and the poly(ethylene terephthalate) (PET) spacers (3.5 μm thick) were used to maintain the desired thickness and constant geometry of the sample. High-temperature FTIR measurements of nP were conducted in the ATR mode using GladiATR (PIKE Technologies) in the temperature range of 300–373 K. An average of 16 scans with the resolution of 4 cm^–1^ was collected in the wavenumber range of 400–4000 cm^–1^. A transmission solution cell with KBr windows (the path length of 1.04 mm) was used to obtain FTIR spectra of XAs solutions in benzene and cyclohexane (0.1 and 0.01 M). To perform the deconvolution of the OH stretching vibration band, MagicPlot Pro software (version 2.9.3, MagicPlot Systems LLC, Saint Petersburg, Russia) was used. The step-by-step process of the deconvolution procedure is described in Reference.^28^

### 2.3 Raman Spectroscopy

The Raman measurements for 3Cl1P and its 0.1 M solution in cyclohexane were performed using a Horiba Xplora Plus Raman spectrometer with a laser operating at 780 nm (the power 30 mW). The Olympus MPlanN 10× objective was chosen. Every spectrum was recorded with an acquisition time of 2 s and an accumulation of 60 scans.

### 2.4 Molecular Dynamics Simulations (MDS)

Molecular dynamics simulations were carried out using the GROMACS package (version 2020).^31−33^ Interactions in the systems were described using the general AMBER force field (GAFF)^34^ and topology provided by AmberTool21.^35^ The simulation parameters were adopted the same as in our previous paper.^10^ Structure factors were calculated based on the optimized models of spatial arrangement of molecules (2000 molecules) using of the TRAVIS software.^36−38^

### 2.5 Density Functional Theory (DFT) Calculations

DFT calculations using the B3LYP and CAM-B3LYP functionls, combined with the basic set 6-311G(d,p), were performed in the Gaussian09 software package.^39^ The second functional was used because it improves long-range interactions, which are important for determining the energy and geometry of the dimers. The single molecule’s and dimer’s geometries have been optimized using the opt = tight and int = very tight options. The interaction energy was estimated by using the counterpoise method to correct the base set superposition error (BSSE). Initial single molecule and hydrogen bonded dimer structures were prepared using GaussView 5.^40^ The dipole moment of each molecule was decomposed into components oriented parallel and perpendicular to the straight line passing through the carbon atom (C1) bound to the hydroxyl group and the farthest atom of the chain (C3 for propanol and X = Cl, Br, or I for molecule containing halogen atoms).

### 2.6 Density Measurements

The density (ρ) of nP, 3Cl1P, and 3Br1P was determined using a vibrating-tube densimeter DMA 4500 M (Anton Paar, Austria). The apparatus was calibrated directly before measurements with dry air and bidistilled water. The water was always freshly degassed (by boiling) before using its electrolytic conductivity was 1 × 10^–12^ S·cm^–1^ at *T* = 298.15 K. Importantly, viscosity-related errors were automatically corrected in full range, which was checked using the oil N100 at 293.15 and 323.15 K. Standard uncertainties of ρ and *T* are *u*(ρ) = 0.002·ρ and u(*T*) = 0.01 K, respectively. It was taken into account that the directional coefficients of the linear functions for 3Cl1P and 3Br1P are similar, and an analogous nature of the density change for 3I1P was assumed. The estimated density for 3I1P was obtained.

### 2.7 Refractometry

The refractive index (RI) measurements of the examined liquids were carried out using the Mettler Toledo refractometer RM40 in the temperature range from 303.15 to 353.15 K. The temperature stability controlled with the aid of a built-in Peltier thermostat was better than 0.1 K. The light source is a light-emitting diode (LED), the beam of which passes through a polarization filter, an interference filter (589.3 nm), and various lenses before it reaches the sample via the sapphire prism characterized by a high thermal conductivity. The measurements of RI were performed with a resolution of 0.0001.

### 2.8 Differential Scanning Calorimetry (DSC)

The studied alcohols: 3Cl1P, 3Br1P, and 3I1P were measured calorimetrically with the use of a Mettler-Toledo DSC apparatus equipped with an HSS8 ceramic sensor (heat flux sensor with 120 thermocouples) and a liquid nitrogen-cooling accessory. The temperature-dependent measurements were conducted on the samples previously poured into a sealed aluminum pan of 40 μL volume. The thermograms were collected on cooling and heating in the temperature range of 123–298 K. The cooling and heating rates were 10 K min^–1^, respectively. The calorimetric measurements were carried out in the atmosphere of nitrogen with a flow of 60 mL min^–1^. Glass transition temperature of each compound was determined from the heating scans as the midpoint of the glass transition step.

## 3 Results and Discussion

As a first step, we performed dielectric measurements in a wide range of temperatures to check whether the D relaxation exists in the halogen derivatives and to what extent its properties are similar to what we observed in *n*-propanol. Representative loss spectra ε″(*f*) of nP obtained at selected temperatures above the glass transition temperature (*T*_g_), revealed the presence of two relaxation processes (Figure S1). The dominant D mode and α relaxation (detected as an excess wing on the high frequency (*f*) flank of the D process) of smaller amplitudes can be distinguished. The secondary relaxation (β) also occurs in nP,^11^ but is not described in this publication. In the case of halogen derivatives of nP, the dielectric loss spectra are more affected by the contribution of the direct current (dc) conductivity (σ), which partially covers the dominating D process. Stronger conductivity contributions in the spectra in halogen derivatives of nP can be explained by more ionic impurities that could originate during the obtaining processes of alcohol. This effect is well illustrated in the dielectric loss spectra of 3Cl1P; please see Figure 1b. In order to better visualize the maxima of the detected relaxation processes, we subtracted a conductivity contribution from the raw data. Figures 1c,d show a comparison of ε″(*f*) for nP and 3Cl1P, measured at selected temperatures after subtraction of the conductivity, together with the fits utilizing the Debye and Cole–Davidson functions. One can see that for 3Cl1P, the α relaxation process becomes more prominent with respect to nP.

To illustrate this effect, we compared the loss spectra characterized by the same peak frequency of the D relaxation (Figure 1e). This enabled us to demonstrate that the amplitude of the D process decreases, whereas the intensity of the structural relaxation increases in the case of XAs compared to that of nP. Moreover, as the atomic mass of the electronegative X atom decreases, the structural relaxation intensity increases. To parametrize the difference between alcohols we compare the ratios of ε″_max_. Debye/ε″_max_. structural and received: nP (19.65), 3Cl1P (1.57), 3Br1P (1.87), and 3I1P (2.89). The work of Gainaru and co-workers^26^ has shown that the structural relaxation in alcohols comes from the movements of the alkyl chains. In the case of halogen alcohols, the dipole moment of the chain is larger; this is due to the presence of halogen atoms. Thus, the apparent amplitude of structural relaxation is larger. Hence, the addition of the moiety characterized by varying polarity to the nonpolar part of the molecule should lead to a change in the amplitude of the primary relaxation. In fact, this is what we observed in our experiment. Thus, these experimental data can be treated as additional evidence supporting the TCM and the molecular origin of the D process in the self-assembled clusters.

In addition, we detected a change in the glass transition temperature (*T*_g_) as well as in the position of the D process in the studied herein systems (see details in the Supporting Information). These findings can be well visualized by comparison of the dielectric spectra measured at similar temperatures in one chart (inset in Figure 1e). From this figure, one can find that all recorded variations scale up with the size of the X atom. Therefore, the 3I1P with the largest mass shows the greatest shift toward the low frequencies of the D process and the highest *T*_g_.

**Figure 2:**
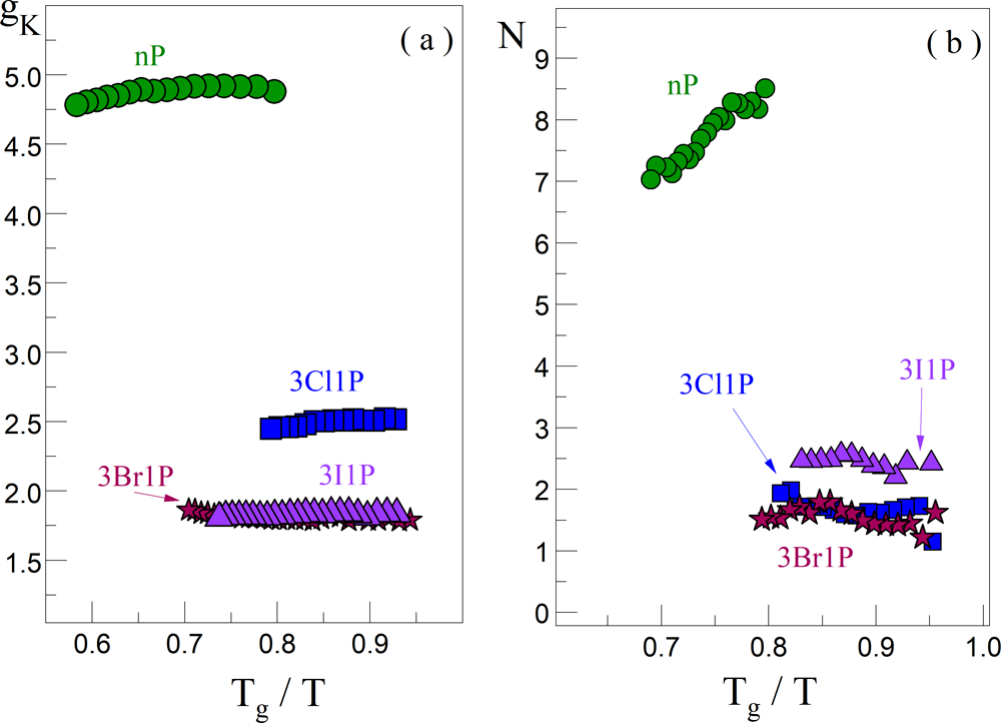
Kirkwood–Fröhlich factor for the studied alcohols (a). The number of molecules creating the transient chains is the source of the Debye relaxation process according to the transient-chain model (b).

The variations described above in the *T*_g_ and amplitude of the Debye process indicate that the presence of X atoms influences the self-association process in the measured samples. To verify this hypothesis, we decided to calculate the Kirkwood–Fröhlich factor (*g*_*k*_), which is a useful parameter allowing us to get an insight into the long-range correlation between dipoles induced by the self-association process.^41,42^ For the computation of *g*_*k*_, the following equation was used:
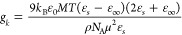
1where ε_s_ and ε_*∞*_ are the static and high-frequency permittivity, respectively; *N*_A_ is Avogadro’s number; ρ is the density of the liquid at temperature *T*; μ_0_ is the dipole moment of the isolated molecule; ε_0_ is the absolute permittivity of vacuum; *M* is the molecular weight; and *k*_B_ is Boltzmann’s constant.^41^ As observed in Figure 2a, the determined values of *g*_*k*_ are much higher than 1 for all studied samples, suggesting that rather chain-like structures are formed, irrespective of the sample. In the vicinity of 169 K, the highest *g*_*k*_ coefficient is obtained for nP (*g*_*k*_ ≈ 4.7), the next for 3Cl1P (*g*_*k*_ ≈ 2.5), and the lowest one (*g*_*k*_ ≈ 1.8) is for 3Br1P and 3I1P. Therefore, similar to the change in the *T*_g_ or in amplitude of the Debye process, the variation in the *g*_*k*_ scales up with the molecular weight and polarity of the halogen atom. Nevertheless, it should be noted that the highest *g*_*k*_ determined for nP suggests the most prominent long distance correlations between dipoles in this alcohol.^43^

Having in mind the above discussion, we further estimated the average number of molecules involved in the formation of the transient chains (*N*) based on the formula proposed by Gainaru and co-workers^26^ (see details in the Supporting Information). In Figure 2b, *N* as a function of *T* was presented. As can be seen, the largest transient chains are formed by nP (*N* = 7–8 molecules). On the contrary, XAs show lower numbers of the molecules involved in the formation of the H-bonded clusters: 2–3 molecules for 3I1P and 1–2 molecules for 3Br1P as well as for 3Cl1P. Thus, it should be noticed that, according to the TCM, the longest H-bonded oligomers among XAs are created in the alcohol bearing the heaviest X atom in its structure. It is a nonintuitive finding since one could expect that the largest X atom introduces the most serious hindrance preventing clustering. What is even more surprising is that the change in *N* is at odds with the variation of *g*_*k*_ in halogen derivatives of nP. These unexpected results of the dielectric investigations forced us to perform additional FTIR studies supported by MD simulations to address the observed peculiarities.

**Figure 3:**
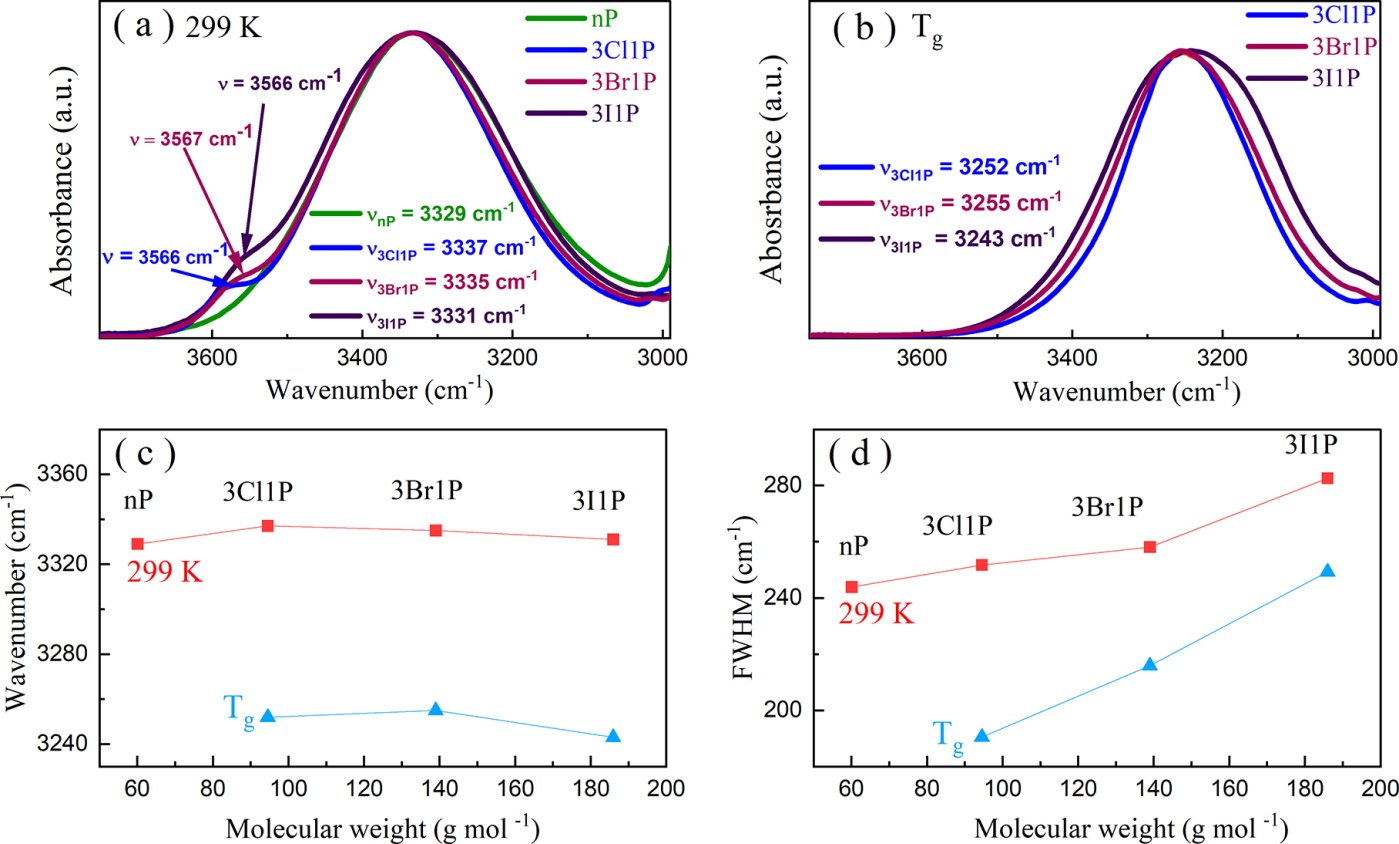
FTIR spectra of alcohols in the frequency range of 3750–3000 cm^–1^ measured at (a) 299 K and (b) glass-transition temperature (*T*_g-3Cl1P_ = 142 K, *T*_g-3Br1P_ = 150 K, and *T*_g-3I1P_ = 157 K). The spectra were normalized to the maximum intensity of the OH stretching vibration band. (c) and (d) Frequency and full width at half-maximum (fwhm) dependencies of the OH stretching vibration band as a function of the molecular weight of XAs, respectively.

The temperature-dependent FTIR spectra obtained for all examined systems are presented in Figure S5. The representative spectra at room temperature around 299 K and at *T*_g_, in the spectral range of 3750–3000 cm^–1^, are shown in Figure 3a,b. The FTIR spectra at room temperature show two characteristic bands: at ∼3330 cm^–1^ and ∼3560 cm^–1^, which are assigned to the stretching vibrations of H-bonded (ν_*OH*_^*HB*^) and non-H-bonded (free) hydroxyl groups (ν_*OH*_^*free*^), respectively. The band at 3560 cm^–1^ is absent in the spectrum of nP at room temperature and *T*_g_ (compare with Figure 3a,b), indicating its complete association by HBs. On the other hand, 3I1P is characterized by the highest intensity of the ν_*OH*_^*free*^ band, while 3Cl1P is characterized by the lowest one, at 299 K. This fact can be connected with the atomic radius of halogens, i.e., as the size of the X atom (the steric hindrance) increases, the degree of association of XAs via O–H···O bonds decreases. Additionally, the percentage of non-hydrogen-bonded hydroxyl groups in 3Cl1P, 3Br1P, and 3I1P at room temperature, calculated from the analyzed spectra, is equal to 2.15%, 2.36%, and 3.13%, respectively. The presence of ν_*OH*_^*free*^ band was also confirmed through FTIR measurements of the studied alcohols in nonpolar solvents, i.e., cyclohexane and benzene (see Figure S6). As shown in Figure S6, the type of solvent influences the self-assembly process of propanols under investigation, i.e., the different ν_*OH*_^*free*^ band position and intensity ratio of the ν_*OH*_^*free*^ and ν_*OH*_^*HB*^ bands for the same alcohol concentration. Moreover, based on Figure 3c, one can also see that the analyzed alcohols do not differ significantly in the position of the ν_*OH*_^*HB*^ band, which indicates a similar strength of formed HBs at room temperature. During cooling, the red shift of the ν_*OH*_^*HB*^ band occurs, demonstrating the strengthening of H-bonding interactions between XA molecules. The ν_*OH*_^*HB*^ bandwidth (full width at half-maximum, fwhm) reduces when temperature decreases (Figure 3d). Such an effect suggests the formation of a more homogeneous network of HBs at lower temperatures. These temperature-induced spectral variations of the ν_*OH*_^*HB*^ bands are similar to those observed for the other Mas.^44^ Interestingly, the ν_*OH*_^*HB*^ bandwidth increases with the increasing weight of the alcohol molecule at both room temperature and *T*_g_ (Table S3). Thus, one can state that the increasing steric hindrance due to change in the size of X atom (Cl, Br, and I) in alcohol molecules causes a more heterogeneous distribution of the HBs’ strength. Alternatively this effect can be a manifestation of the halogen based H bonding in the derivatives of nP. To address this hypothesis, we also performed Raman measurements for 3Cl1P and its 0.1 M solution in cyclohexane and compared them with the IR results (Figure S7). It should be noted that according to DFT calculations, the peak originating from the stretching vibration of free C–Cl group in cyclohexane occurs at 698 cm^–1^, while the one from the H-bonded C–Cl group in the dimer is located at 682 cm^–^^1^. As can be seen in Figure S7, the peak of the C–Cl stretching vibration in diluted 3Cl1P is observed at 662 cm^–1^ (IR) and 661 cm^–1^ (Raman), whereas in bulk, the band’s position occurs at 656 cm^–1^ (both for IR and Raman spectra). Thus, the weak shift in these band positions (Δν(IR) = 6 cm^–1^, Δν(Raman) = 5 cm^–1^ observed for the spectra of bulk and diluted 3Cl1P may indicate that the Cl atoms participate in different intermolecular interactions including halogen–halogen and halogen based H bonds. However, due to their weak strength, the change in C–Cl stretching vibration is very small.

Further, the activation enthalpy (*E*_*a*_) of the dissociation process of alcohols was calculated based on the van ’t Hoff equation, according to the procedure described in our previous paper (see Figure S8).^28,45^ The *E*_*a*_ values demonstrate a significant drop for XAs (from 15.2 kJ·mol^–1^ for 3Cl1P to 7 kJ·mol^–1^ for 3I1P) compared to that for nP (33.8 kJ·mol^–1^). This can be simply explained having in mind that the presence of the X atom prevents the alcohol molecules from linking into larger aggregates by HBs, as deduced from dielectric investigations. As a consequence of that, the effect of cooperativity of these specific interactions gets weaker. Alternatively, one can also suppose that halogen based hydrogen bonds (manifested as growing ν_*OH*_^*HB*^ bandwidth and weak shift in ν_*Cl*_) appear in the studied derivatives of nP. Nevertheless, to confirm this hypothesis and gain a much deeper insight into the structure of the studied herein alcohols, further molecular dynamics simulations were performed.

**Figure 4:**
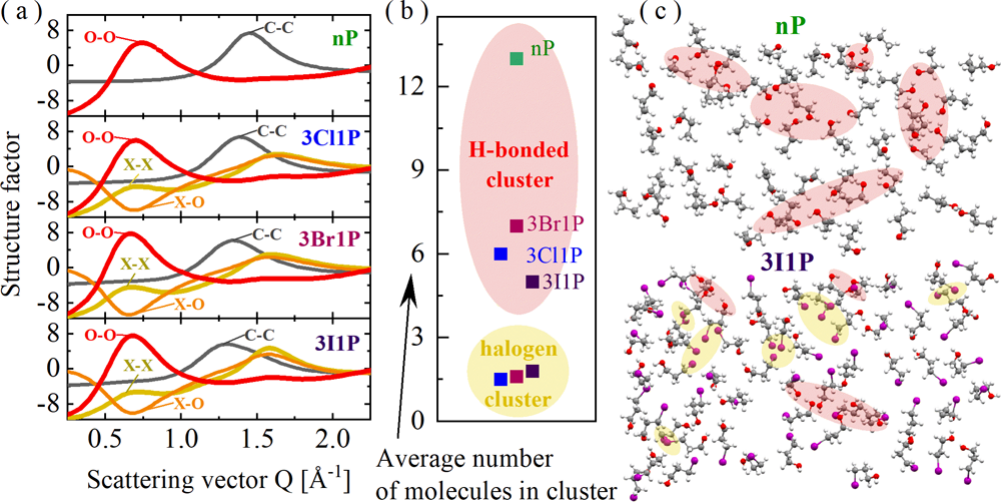
Results obtained from molecular dynamics simulations: partial structure factors (a), the average number of molecules in H-bonded and halogen clusters (b), and 2D fragments of the structural models for nP and 3I1P demonstrating the clusters of OH groups and halogen atoms (marked in red and yellow, respectively) (c).

MDS provided a more illustrative description of the supramolecular associates in the investigated compounds. The structural model of each alcohol was optimized based on the system of 2000 molecules at room temperature using a general AMBER force field (GAFF) in the Gromacs package. The model-based structure factors, which give information about the atom–atom spatial correlations, are depicted in Figure 4a. The data are shown in the range of up to 3.0 Å^–1^ where the intermolecular interactions play the major role. Only selected partial structure factors are presented for clarity–those having the biggest contribution to the intermolecular correlations (C–C, O–O, and X–X). All partial functions can be seen in Figure S11a. The sum of all partial functions, where C, O, H, and X refer to carbon, oxygen, hydrogen, and halogen elements, respectively, after multiplication by respective atomic form factors and weight fractions, is called the total structure factor *S*(*Q*). For nP and 3Cl1P, the total *S*(*Q*) was also derived experimentally from the X-ray scattering data (*S*(*Q*) for 3Br1P and 3I1P was not possible to determine using the laboratory diffractometer due to high absorption and fluorescence). The experimental and model-based *S*(*Q*) values for nP and 3Cl1P show good agreement (see Figure S12), validating the accuracy of the performed simulations and analyzed models.

The main *S*(*Q*) peak at scattering vector *Q* around 1.3–2.0 Å^–1^ arises due to nearest-neighbor spatial correlations between molecules, and the principal contribution to this peak is given by C–C correlations of alkyl tails. Whereas the organization of molecules in bigger associates, which constitute the microstructure of the liquids, yields peaks at *Q*-values below 1 Å^–1^. The main contribution to this organization for nP and all XAs is given by the O–O correlation at around 0.7 Å^–1^ (Figure 4a). This peak is an evidence for the creation of supramolecular clusters where molecules are organized through OH groups. The O–O distribution function (shown in Figure S11b) induces a large first peak with a maximum at around 2.8 Å, extending up to around 3.5 Å, which is a direct consequence of the structuring of O atoms in O–H···O bonds. Moreover, the pair distribution functions for XAs reveal that there are X-ray-induced O–X distances starting from around 3.0 Å and X–X distances with a clear maximum at around 3.5–4.0 Å (the bigger the halogen atom, the greater the X–X distance). The O–X and X–X oscillations extend up to around 15–20 Å (shown in Figure S11c), which indicates the creation of the medium-range order by these groups. As a consequence of that, X–X correlations also give a maximum in the *S*(*Q*) at around 0.7 Å^–1^ (Figure 4a) and suggest that halogen atoms of neighboring molecules may also group into small aggregates; these are called here as “halogen clusters”. In turn, the O–X correlations give minima (antipeaks) in the *S*(*Q*) at around 0.7 Å^–1^ and indicate that X atoms at one end of the molecular tail are anticorrelated with OH groups at the second end; there is a segregation between these atomic groups. More information on the origin of the *S*(*Q*) in alcohols may be found here.^46,47^

Taking into account the structural correlations between atoms and their distances, we distinguished two types of clusters formed in the studied systems: “H-bonded clusters” including the O–O and O–X correlations or “halogen clusters” including the X–X correlations. For both cluster types, we calculated the average number of molecules involved in the aggregates, assuming only a simple condition that a molecule forms a cluster with another one when the distance between the specific atoms is smaller than the cutoff distance of the appropriate radial distribution function: O–O and O–X ≤ 3.5 Å for the H-bonded clusters and Cl–Cl ≤ 3.8 Å, Br–Br ≤ 4 Å, and I–I ≤ 4.3 Å for the halogen clusters. The histograms of the cluster sizes are presented in Figure S13a, whereas the average numbers of molecules in such defined clusters are presented in Figure 4b and show that the biggest H-bonded clusters are formed in nP (∼13 molecules). In turn, much smaller nanoassociates connected by HBs exist in halogen compounds (5–7 molecules on average). It is also worth to mention that the derived histograms and the average number of molecules in the H-bonded clusters for nP are very similar to data estimated by other researchers from various computer simulations for nP as well as other simple linear MAs such as *n*-ethanol and *n*-butanol, at room temperature.^46,48−50^ However, it should be added that they strongly depend on, e.g., the force field choice and the cluster definition. Here, we used a very broad definition with only distance constraints, so the values of the number of molecules in what we call “clusters” may be overestimated, with respect to the ones obtained from the extrapolation data presented in Figure 2b. Hence, the observed strong discrepancies may be due to a much narrower definition for the transient-chain clusters and assumptions of the model used. Moreover, the data derived from MDS provided systems with a slightly too high structural order compared to experimental data, which may also affect the overestimation of the cluster sizes.

The general organization of molecules in the MD models and the clusters of OH groups are marked on the representative fragments of models for nP and 3I1P in Figure 4c. Moreover, we were able to identify on the models the clusters of halogen atoms. Such aggregates are rather small (dimeric, trimeric) and the average number of molecules associated with such clusters increases with the bigger mass of the halogen atom (1.5 for Cl, 1.6 for Br, and 1.8 for I). The subtle balance between the conventional hydrogen bonding between OH groups and other interactions involving the halogen atoms in the studied relatively simple molecular systems appears to drive very complex heterogeneous microstructure. It is also important to note that we found great agreement of the MDS with the outcomes of the spectroscopic studies exhibiting the lower degree of association of molecules via O–H···O bonds in XAs compared to ordinary nP. The percent of non-H-bonded molecules, determined from the histograms of the cluster sizes derived from MDS, is 1.8% for nP and 3.6, 4.3, 6.4% for 3Cl1P, 3Br1P, and 3I1P, respectively. Thus, it is also in accordance with the FTIR results: The fraction of free molecules increases in the same manner as the intensity of the ν_*OH*_^*free*^ band in IR spectra. One more property of these systems derived from MDS that is consistent with the IR outcomes is a very similar distribution of the O–O lengths in HBs, with the maximum located at around 2.8 Å for all alcohols at room temperature (shown in Figure S13b), suggesting a similar strength of HBs in nP and XAs, despite attaching the halogen atom.

Since experimental and MDS data discussed above indicated that there are different specific interactions including halogen–halogen, halogen-based, and classical H-bonds in the studies systems, additional DFT calculations were applied to evaluate the energy of such interacting systems. Based on the analysis of the geometry of different dimers that were considered, we can conclude that strong hydrogen interactions of the O–H···O type (interaction energy *E* ≥ 5 kcal/mol) predominate in the studied alcohols. H–O···X interactions are weaker, but still possible (*E* < 4 kcal/mol), whereas the X···X forces between halogen atoms seem to be the weakest (*E* < 2 kcal/mol). A detailed analysis of the dimeric structures and their interaction energies based on the DFT calculations is included in the Supporting Information. It is worth mentioning that DFT models yield very similar O–O distances in HBs for all studied alcohols (2.9 Å, see Figure S6) to those obtained from MDS (2.8 Å). Also, the O–X and X–X distances are consistent in both theoretical methods, DFT, and MDS, which authenticates the analyzed models of the halogen alcohols.

## 4 Summary

Summarizing the data discussed in this letter, one can deduce that strongly electronegative halogen atoms: Cl, Br, and I have a significant impact on the molecular association processes. Our studies have shown that molecules in *n*-propanol and its halogen derivatives tend to form clusters via hydrogen bonds during the vitrification process, and the architecture of the HBs in such clusters is rather chain-like. The introduction of halogen atoms into the alkyl chain significantly inhibits the association of molecules by HBs, which is revealed by the greater amount of nonassociated molecules and smaller size of the H-bonded clusters in halogen derivatives compared to pure *n*-propanol. Moreover, based on experimental spectroscopic studies and theoretical calculations we found some strong indications that other types of small molecular associates can be formed due to O–H···X or X···X interactions, where X indicates the halogen atom. They compete with the O–H···O forces and introduce local disorder and heterogeneity into the supramolecular structure. Therefore, these two important findings can be used to explain why the change in the Kirkwood–Frölich factor with respect to the number of molecules in the transient chain model can be an explanation. It is also worth to stress that very weak O–H···X or X···X interactions to contribute to the enormous drop of the dissociation enthalpy of HBs in the investigated halogen derivatives of *n*-propanol, despite of the fact that the position of the stretching vibration of OH group as well as the length of H-bonds remain unaffected by the structure of the molecule differing in the presence and type of the halogen atom. Finally, our data also provide experimental evidence of the molecular origin of the D relaxation process in self-assembling alcohols. Here, we show the relationship between the association of the tested alcohols into chain systems (first degree of association) and, for example, the Kirkwood coefficient and Debye relaxation amplitude. According to them, the largest *g*_*k*_ and Debye process is, in the case of the most associative alcohol, that is, nP. On the other hand, the presence of halogen atoms and the formation of other supramolecular structures (other than those formed through OH–O bonds, among other things) caused by them is the reason for the lower intensity of Debye relaxation in halogen alcohols. We are convinced that the obtained results will contribute to a much better understanding of the self-assembly process in highly viscous systems.

## Supporting Information Available

Details of fitting dielectric spectra with use of Havriliak–Negami functions, calculations of average cluster size of alcohol aggregates, temperature-dependent FTIR spectra and analysis of the studied alcohols, details of the molecular dynamics simulations, dimer interaction energy calculations (PDF)
