# Supplementary material for: Experimental and Computational Approach to Studying Supramolecular Structures in Propanol and Its Halogen Derivatives
Source: J Phys Chem B. 2023 Oct 17;127(42):9102–10. doi: 10.1021/acs.jpcb.3c02092 (PMC10614193; doi:10.1021/acs.jpcb.3c02092)
Supplement: Supplementary file 1 — jp3c02092_si_001.pdf [file jp3c02092_si_001.pdf]

Supporting information

Experimental and Computational Approach to Studying Supramolecular Structures in Propanol and its Halogen Derivatives

Kinga Łucak[a]*, Anna Z. Szeremeta[a], Roman Wrzalik[a], Joanna Grelska[a], Karolina Jurkiewicz[a], Natalia Soszka[b], Barbara Hachuła[b], Daniel Kramarczyk[a], Katarzyna Grzybowska[a], Beibei Yao[a], Kamil Kamiński[a], Sebastian Pawlus[a].

[a] *Institute of Physics, Faculty of Science and Technology, University of Silesia in Katowice, 75 Pułku Piechoty 1, 41-500 Chorzów, Poland*

[b] *Institute of Chemistry, Faculty of Science and Technology, University of Silesia in Katowice, Szkolna 9, 40-006 Katowice, Poland*

*Correspondence e-mails: kinga.lucak@us.edu.pl

1.1. Dielectric data analysis

Figure S1 shows the dielectric loss spectra of nP. We can distinguish dielectric peaks, shifting with increasing temperature, which indicates the dielectric process. The peaks are broadened, so we presume a superposition of two dielectric processes: Debye and α - process.

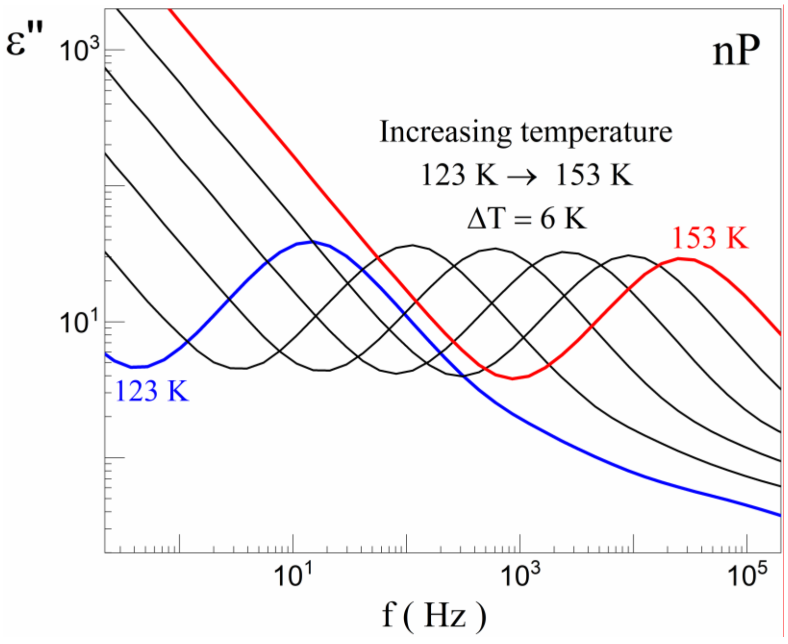

Figure S1. Dielectric loss spectra of nP for selected temperatures.

To properly analyse the observed relaxation processes for nP, the data were fitted with an equation consisting of the sum of Debye, Havriliak−Negami (HN) equation and constant-current conductivity σ_{DC}. The exponents α and β are the parameters for the symmetrical and asymmetrical broadening of the dielectric loss curve, respectively.

$$\varepsilon^*(\omega) = \varepsilon_\infty + \frac{\Delta\varepsilon}{1+\ (i\omega\tau_D)} + \frac{\Delta\varepsilon}{[1+\ (i\omega\tau_{HN})^\alpha]^\beta} + \frac{\sigma_{DC}}{i\omega\varepsilon_0}, \tag{1}$$

Structural relaxation can be described by the HN formula with a parameter α = 1. Which means that the HN equation reduces to the Cole-Davidson function, and finally, the data was being fit by:

$$\varepsilon^*(\omega) = \varepsilon_\infty + \frac{\Delta\varepsilon}{1+\ (i\omega\tau_D)} + \frac{\Delta\varepsilon}{(1+\ i\omega\tau_{CD})^\beta} + \frac{\sigma_{DC}}{i\omega\varepsilon_0}, \tag{2}$$

where ε_∞ is the high-frequency dielectric permittivity, $\Delta\varepsilon$ - the dielectric strength, ω is equal to $2\pi f$, τ_D is the Debye relaxation time and σ_{dc} is the constant-current conductivity [1, 2, 3].

The dielectric loss spectrum of halogen derivatives of propanol is affected by the contribution of the direct current (DC) conductivity σ, which partially covers the Debye process. Two equation does not sufficient to describe the relaxation phenomena at high frequencies. In this case, the data was being fit by: Debye, Cole-Davidson, Cole-Cole functions for the Debye, structural and secondary relaxation, respectively, and constant-current conductivity σ_{DC}[1].

$$\varepsilon^*(\omega) = \varepsilon_\infty + \frac{\Delta\varepsilon}{1+(i\omega\tau_D)} + \frac{\Delta\varepsilon}{(1+i\omega\tau_{CD})^\beta} + \frac{\Delta\varepsilon}{(1+i\omega\tau_{CD})^\alpha} + \frac{\sigma_{DC}}{i\omega\varepsilon_0}, \tag{3}$$

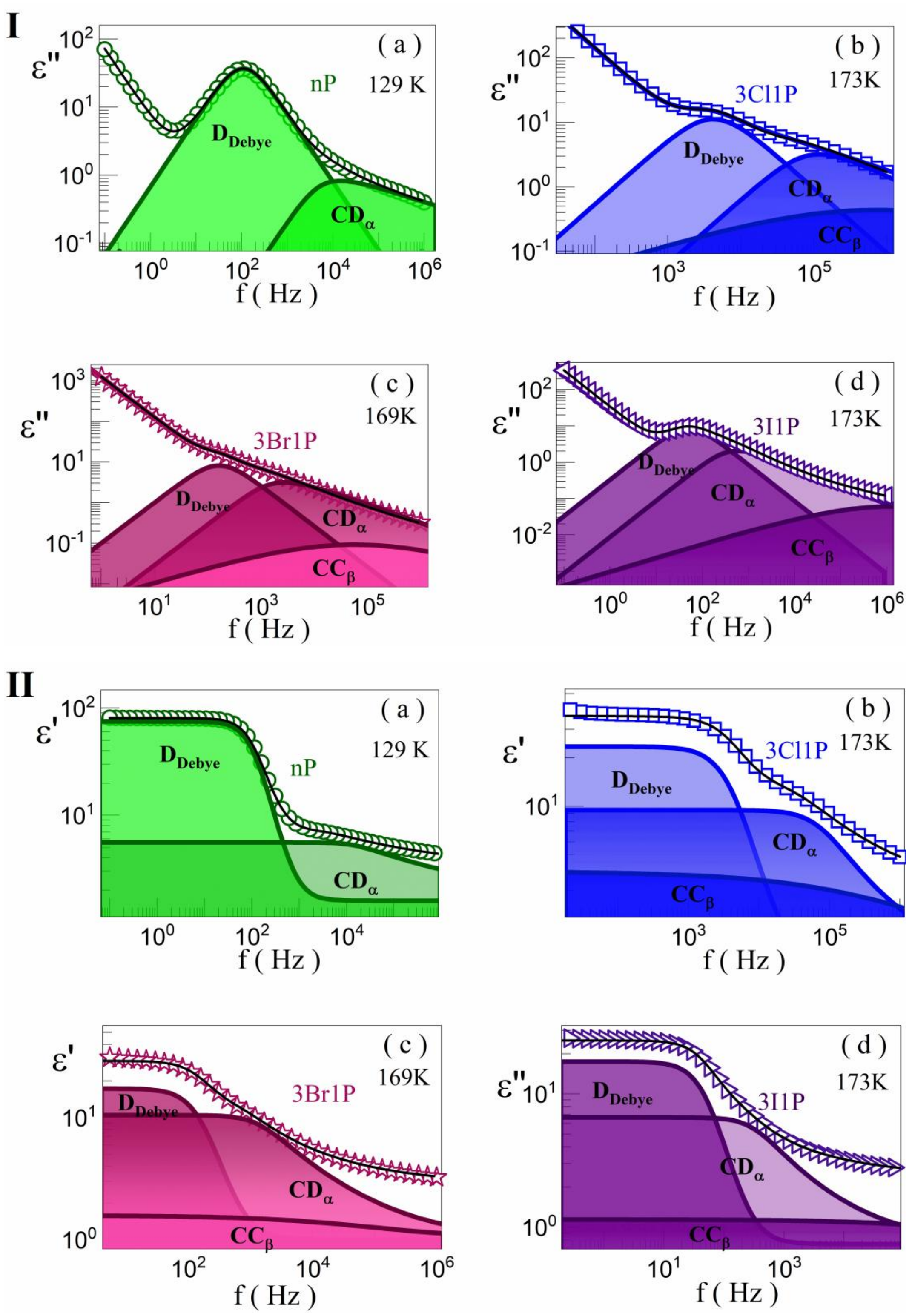

Figure S2. Imaginary (I) and real (II) parts of the dielectric permittivity, obtained for (a) nP at 129 K, (b) 3Cl1P at 173 K, (c) 3Br1P at 169 K, and (d) 3I1P at 173 K. The measurement points are marked with circles (nP), squares (3Cl1P), stars (3Br1P), and triangles (3I1P). The colorful solid lines are the results of fitting the Debye, structural and secondary relaxation

with Debye, Cole-Davidson, and Cole-Cole functions, respectively. The black solid lines show the overall fit lines of the experimental spectra.

To better show the presence of the structural process, we used the Kramers-Kronig transform of the ε’ data[1].

$$\varepsilon''_{der} = -\frac{\pi}{2}\frac{\partial\,\varepsilon'(\omega)}{\partial ln\omega} \approx \varepsilon''_{rel}\ , \tag{4}$$

Below **(Figure S3)** we show the original data at lower temperatures, where the alpha relaxation is better visible. **Figure S4** shows the data obtained after applying the Kramers-Kronig transform.

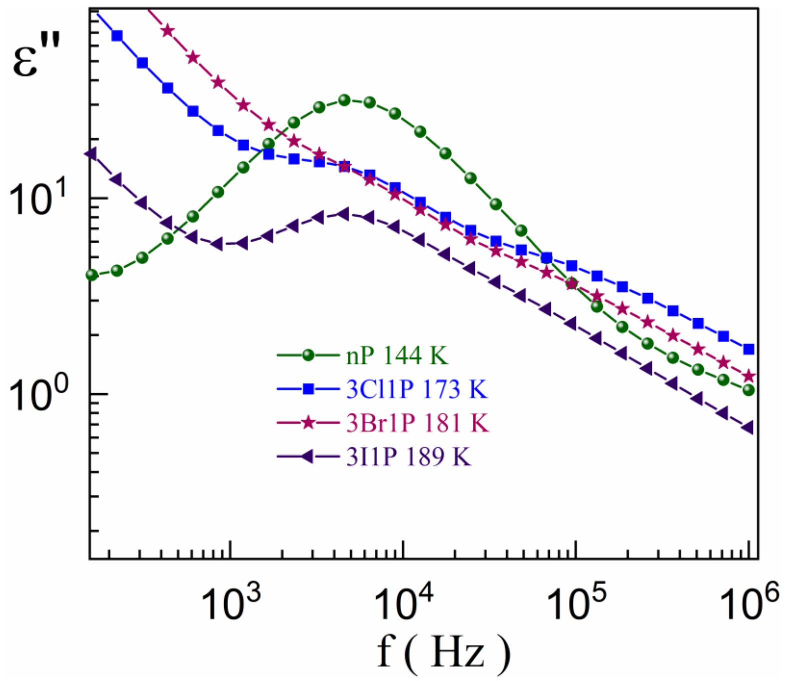

Figure S3. Original data for propanol and its halogen derivatives.

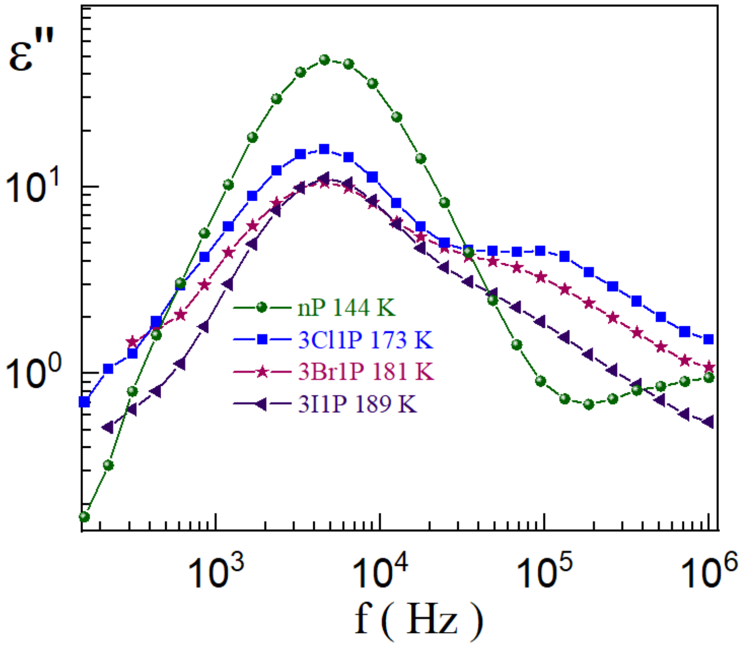

Figure S4. Data obtained using the Kramers-Kronig transform for propanol and its halogen derivatives.

1.2. Calculations of average cluster size of alcohol aggregates.

Gainaru and co-workers [4] developed a formula to estimate the average number of molecules involved in transition chain formation in monohydroxy alcohols. The Gainaru model works very well for alcohols with a hydroxyl group at the end of the carbon chain, such as butanol. In the case of monoalcohols with a halogen atom, to apply the model proposed by Gainaru, we had to take into account the dipole moment coming from the halogen atom. Using the collected data from dielectric measurements and the relation proposed by Gainaru [4], we calculated the number of molecules involved in the self-organisation into clusters via hydrogen bonds (N). Gainaru applied this model based on the fact that the dipole moment of A-type polymers is arranged parallel to the chain backbone [5]. This assumption leads to the following expression [4]:

$$\frac{\Delta\varepsilon_D}{\Delta\varepsilon_\alpha} = \frac{\left(\frac{\mu_{end-to-end}}{\mu\perp}\right)^2}{N} \approx 4N, \qquad (5)$$

where $\Delta\varepsilon_D$, $\Delta\varepsilon_\alpha$ are dielectric strength of the Debye and structural relaxation process, $\mu_{end\text{-}to\text{-}end}$ is of the order of $N\mu$ and $\mu_\perp$ should be $\mu/2$. nP is a simple alcohol with an OH group at the end of the chain, therefore we could implement the Gainaru model for this monoalcohol. In the case of halogen derivatives of propanol, we have to take into account atoms attached to the

3rd carbon atom: Cl, Br and J. Halogen atoms contribute to a change in the dipole moment of the molecule. In propanol μ_{OC} is according to the assumption proposed by Gainaru, while in the case of halogen derivatives of propanol these are respectively: $\mu_{\perp} \approx \mu_{OCl}$, $\mu_{\perp} \approx \mu_{OBr}$, $\mu_{\perp} \approx \mu_{OI}$. (look up **Table S1**).

Table S1. Dipole moments and its components parallel and perpendicular to the cluster axis, calculated for single molecule using the B3LYP/6-311G (d, p) model.

Molecule	μ [D]	$\mu_{\perp}$ [D]	$\mu_{\parallel}$ [D]
1-propanol	1.501	0.825	1.254
3-chloro-propanol	3.269	2.122	2.486
3-bromo-propanol	3.322	2.263	2.432
3-iodo-propanol	3.175	2.142	2.343

Furthermore, the value of the perpendicular component of the molecule's dipole $\mu_{\perp}$ is comparable to the parallel component $\mu_{\parallel}$, while in the case of propanol the parallel component $\mu_{\parallel}$ is twice as large as the perpendicular component $\mu_{\perp}$. Obtaining $\Delta_{\varepsilon D}$ and $\Delta_{\varepsilon\alpha}$ from the spectra of dielectric measurements and assuming that in $\mu_{\perp}$ we take into account a strongly electronegative atom attached at the end of the alkyl chain, we can determine the number of aggregating molecules in the supramolecular chain of halogen derivatives of propanol. The clusters has been calculated using the equation:

$$N = \left(\frac{\Delta\varepsilon_D}{\Delta\varepsilon_\alpha}\right)\left(\frac{\mu_{\perp}}{\mu_{\parallel}}\right)^2. \tag{6}$$

The N values for the analyzed alcohols are presented in **Table S2**.

Table S2. Temperature dependence of the number of molecules in the cluster for studied alcohols, calculated based on the Gainaru model [4].

nP

T [K]	ε_D	ε_α	$\varepsilon_D/\varepsilon_\alpha$	N
123	77.34	3.93	19.65	8.5
125	75.6	3.95	19.15	8.3
127	74.62	3.91	19.09	8.3
129	73.54	3.96	18.45	7.9
131	71.96	3.92	18.35	7.9
133	70.57	3.97	17.76	7.7
135	69.07	4.06	16.99	7.4
137	68.21	4.04	16.90	7.3
139	66.59	3.99	16.69	7.3
141	65.56	3.91	16.76	7.3

3Cl1P

T [K]	ε_D	ε_α	$\varepsilon_D/\varepsilon_\alpha$	N
149	24.48	15.55	1.57	1.1
151	27.84	11.76	2.36	1.7
153	27.42	11.72	2.33	1.7
155	26.76	11.75	2.27	1.6
157	25.96	11.69	2.22	1.6
159	25.37	11.33	2.23	1.6
161	24.97	11.57	2.15	1.6
163	24.82	11.37	2.18	1.6
165	24.49	10.75	2.27	1.7
167	24.05	10.24	2.34	1.7
169	23.26	9.887	2.35	1.7
171	23.02	9.789	2.35	1.7
173	22.83	8.401	2.71	2.0
175	22.44	8.457	2.65	2.0

3Br1P

T [K]	ε_D	ε_α	$\varepsilon_D/\varepsilon_\alpha$	N
157	18	9.61	1.87	1.6
159	16.14	11.45	1.40	1.2
161	17.35	10.39	1.66	1.4
163	16.81	10.25	1.64	1.4
165	16.09	9.75	1.64	1.4
167	16.61	9.98	1.66	1.4
169	16.29	9.46	1.72	1.5
171	16.33	8.81	1.85	1.6
173	16.27	8.5	1.91	1.7
175	15.8	7.62	2.07	1.8
177	15.8	7.62	2.07	1.8
179	15.18	8.15	1.86	1.6
181	14.75	7.46	1.97	1.7
183	14.36	7.46	1.92	1.7
185	14.08	7.96	1.76	1.5
187	14.16	7.91	1.78	1.5
189	13.76	7.89	1.74	1.5

3I1P

T [K]	ε_D	ε_α	$\varepsilon_D/\varepsilon_\alpha$	N
163	17.73	6.12	2.89	2.4
165	17.34	5.954	2.91	2.4
169	16.55	6.278	2.63	2.2
171	16.71	5.91	2.82	2.3
173	16.51	5.788	2.85	2.4
175	16.24	5.48	2.96	2.5
177	16	5.229	3.05	2.6
179	15.64	5.091	3.07	2.6
181	15.43	5.202	2.96	2.5
183	15.21	5.137	2.96	2.5
185	14.84	5.039	2.94	2.5
187	14.6	4.947	2.95	2.5
189	17.73	6.12	2.89	2.4

1.3. Fourier transform infrared and Raman spectroscopy

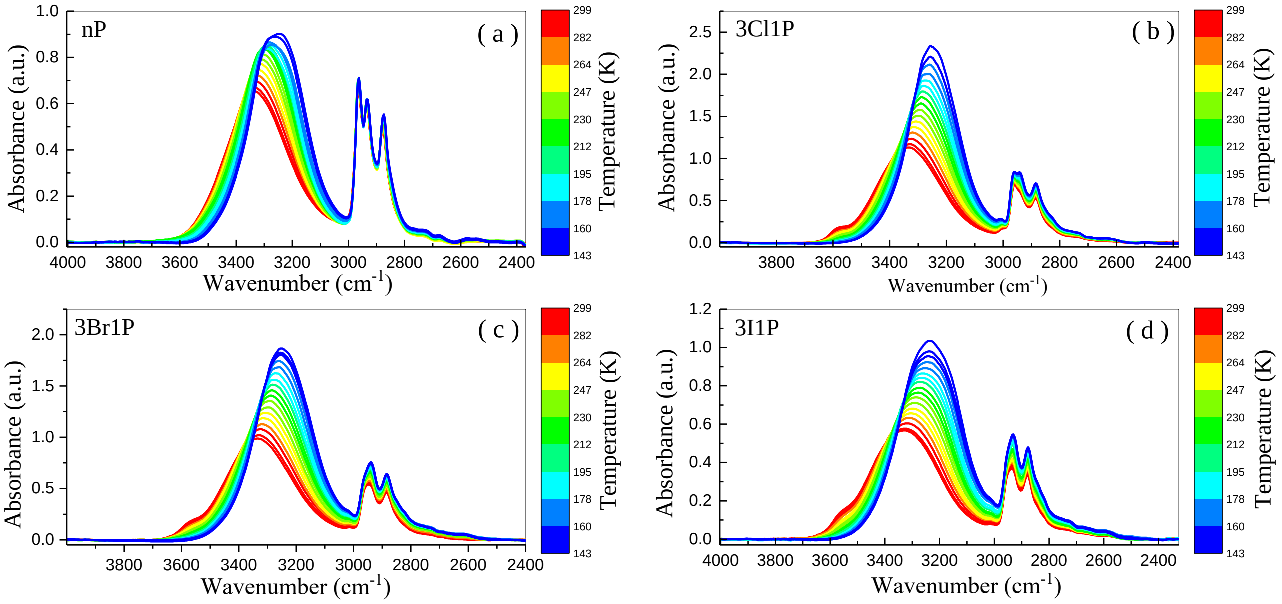

Figure S5. Temperature-dependent FTIR spectra of the analyzed alcohols: (a) nP (T = 299 - 143 K), (b) 3Cl1P (T = 299 - 143 K), (c) 3Br1P (T = 299 - 143 K), and (d) 3I1P (T = 299 - 143 K).

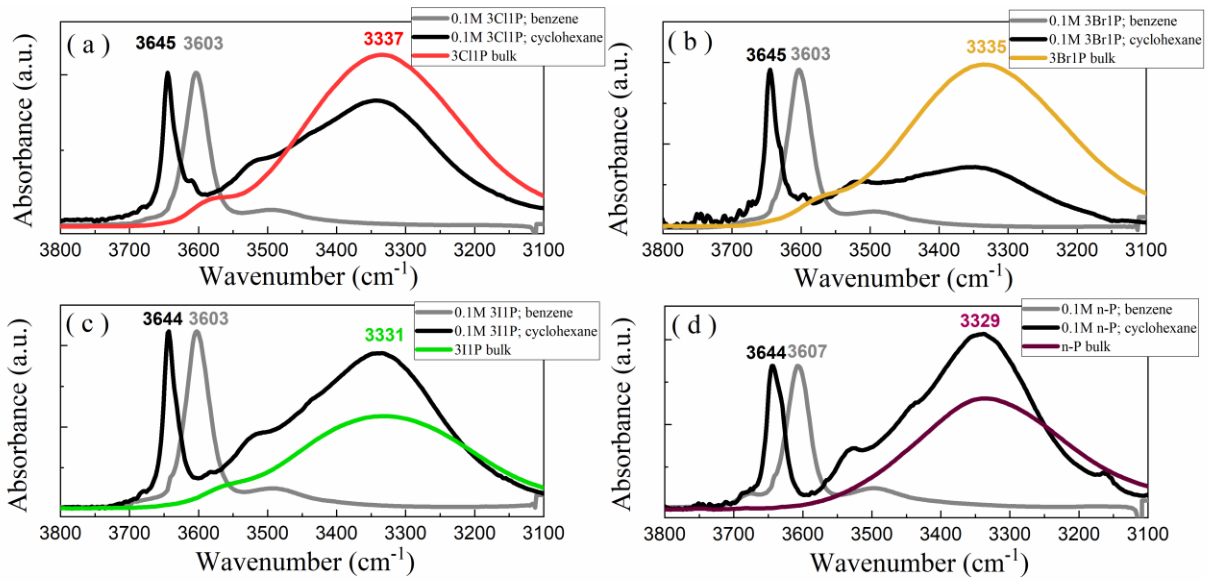

Figure S6. FTIR spectra of alcohols in bulk and dissolved in solvents (benzene, cyclohexane) in the frequency range of 3800 - 3100 cm^{-1}

Table S3. The frequency (ν) and full width at half maximum (FWHM) values of the ν_{OH}^{HB} band for measured alcohols at 299 K and T_g.

Substance	ν (cm^{-1}) at 299 K	ν (cm^{-1}) at T_g	FWHM (cm^{-1}) at 299 K	FWHM (cm^{-1}) at T_g
P	3329	--	243.87	--
3Cl1P	3337	3252	251.71	190.48
3Br1P	3335	3255	258.08	215.85
3I1P	3331	3243	282.6	249.32

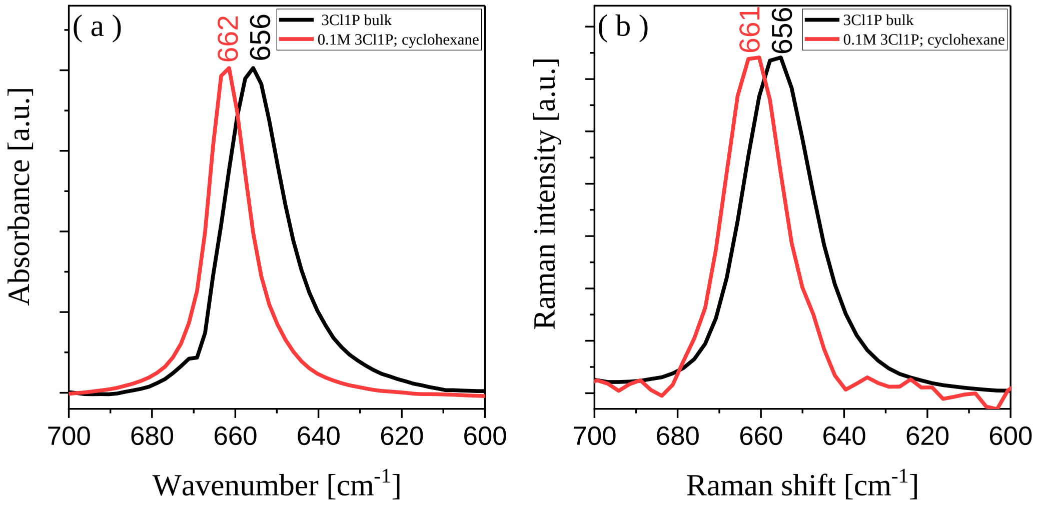

Figure S7. IR (a) and Raman (b) spectra in the frequency region 700 – 600 cm^{-1} presenting the C-Cl stretching vibration band for bulk 3Cl1P (black) and its 0.1 M solution in cyclohexane (red). The spectra were normalized to the C-Cl band intensity.

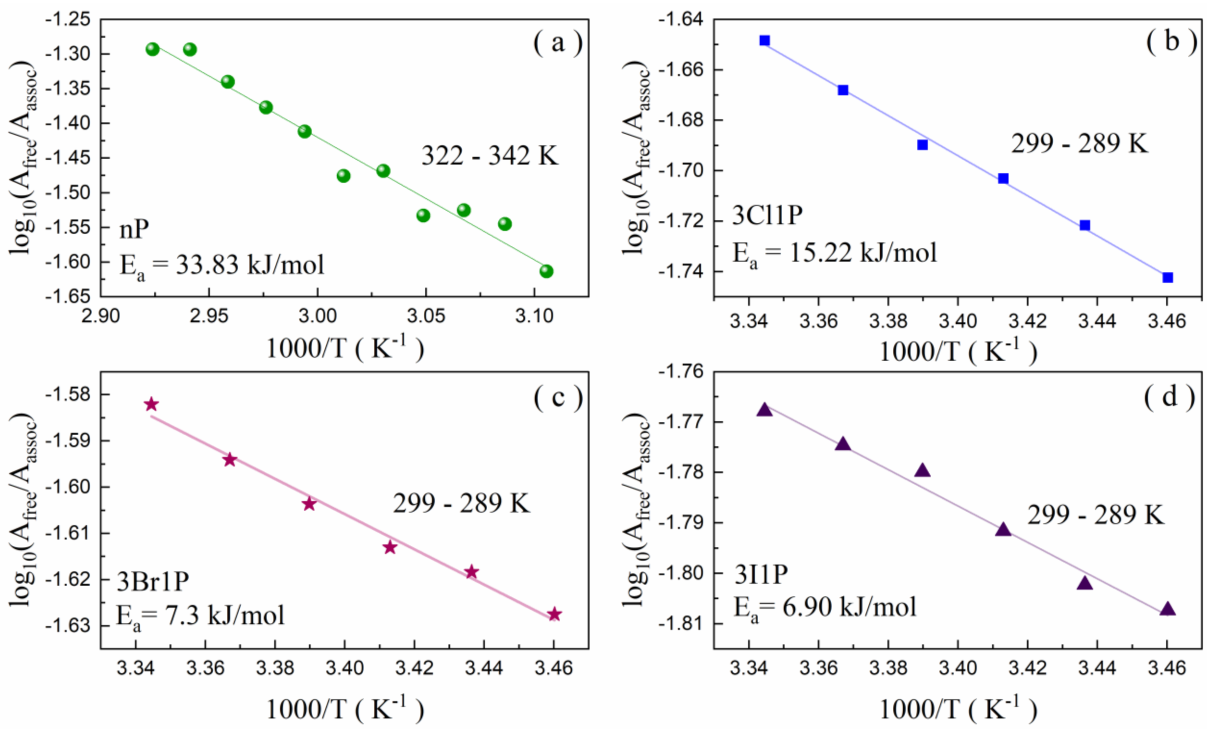

Figure S8. The van't Hoff plots for IR absorption bands of (a) nP, (b) 3Cl1P, (c) 3Br1P, and (d) 3I1P used to obtain the enthalpy of the dissociation process between the H-bonded and free OH species.

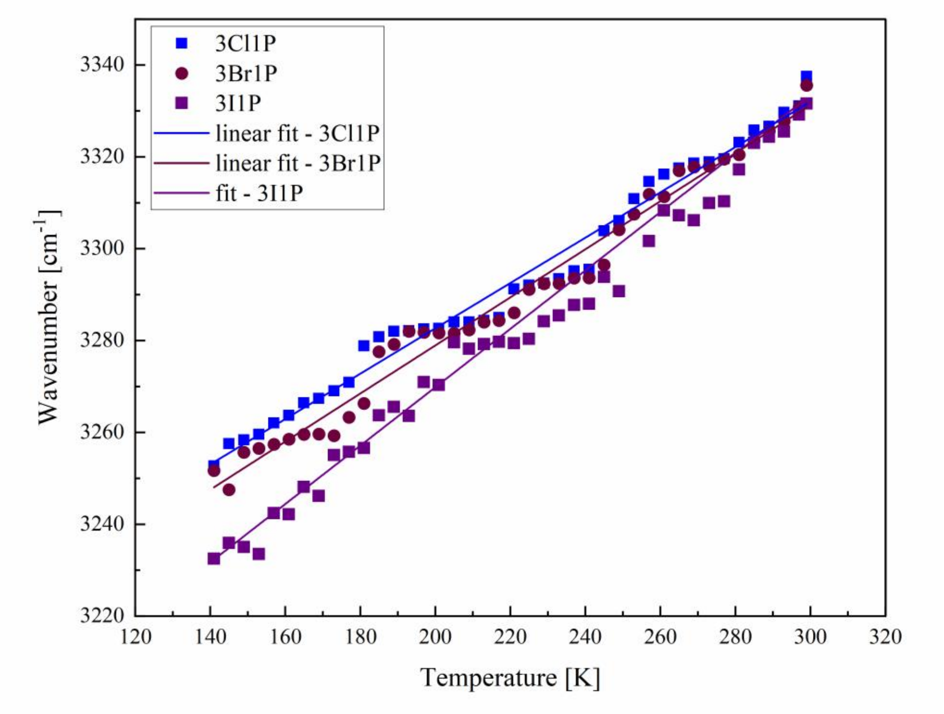

Figure S9. The temperature dependence of $\nu_{OH\ associated}$ peak position for examined halogen derivatives of propanol in the temperature range 299 – 141 K.

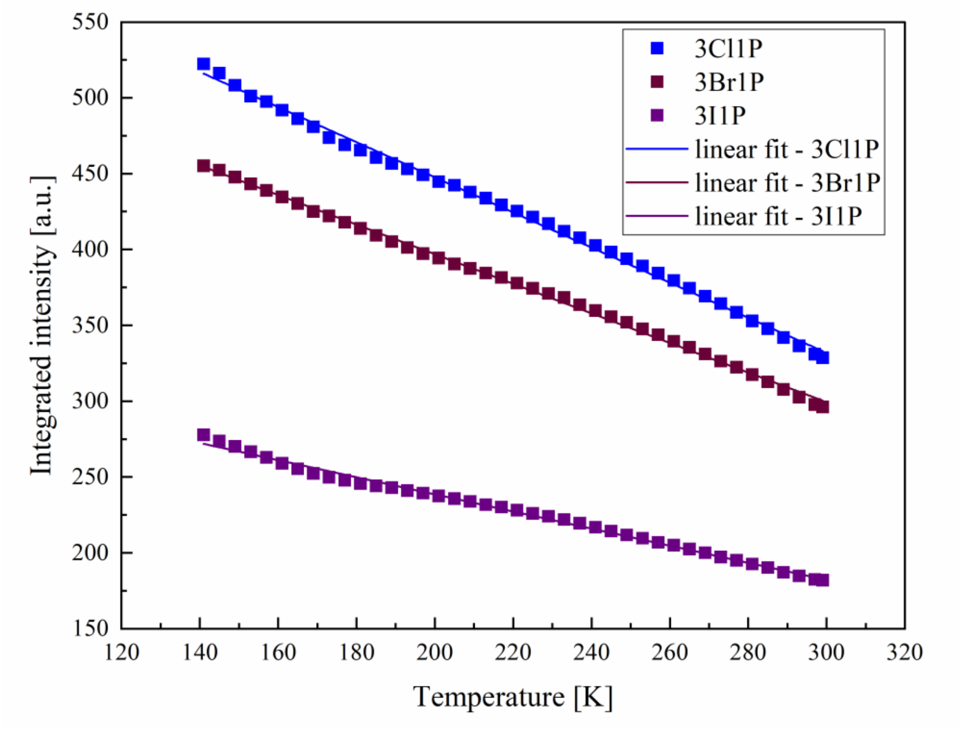

Figure S10. The temperature dependence of the integrated intensity of the ν_{OH} band for examined halogen derivatives of propanol in the temperature range 299 – 141 K.

1.4. Molecular dynamics simulations

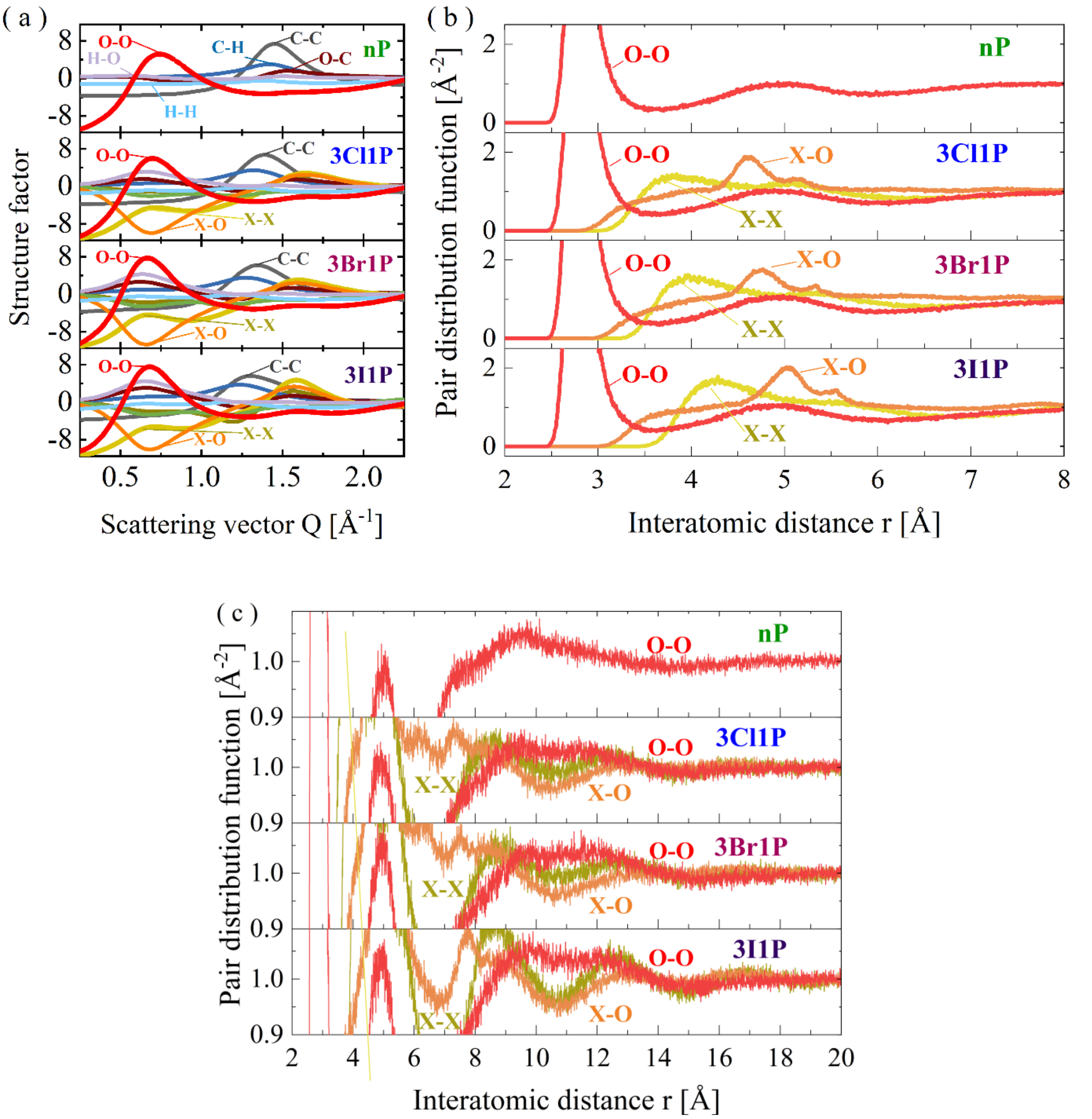

Figure S11. Results obtained from molecular dynamics simulations: all partial structure factors (a) and selected atom-atom pair distribution functions in the range 2−8 Å (b) and 2−20 Å (c). C, O, H, and X = Cl, Br or I refer to carbon, oxygen, hydrogen, and halogen elements, respectively.

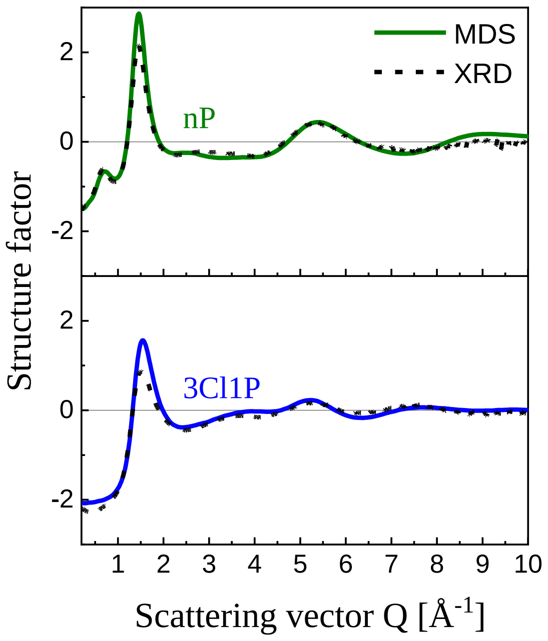

Figure S12. Comparison of experimental total structure factors for investigated nP and 3Cl1P derived from X-ray diffraction measurements (XRD) with theoretical functions derived from molecular dynamics simulations (MDS).

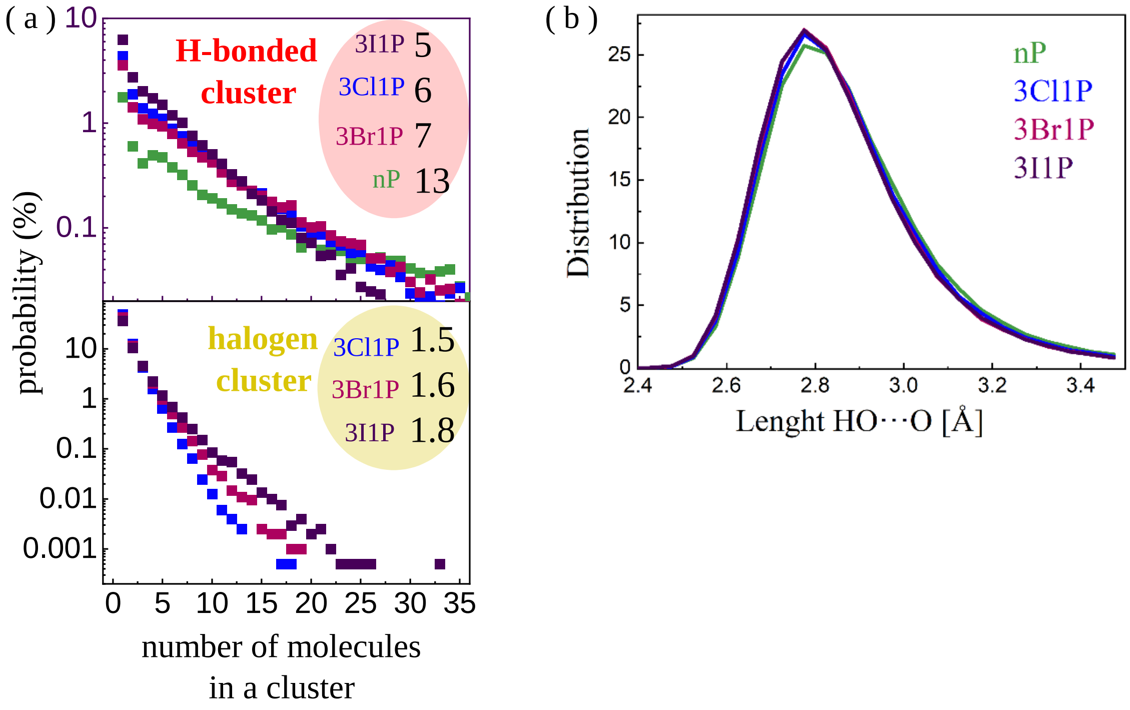

Figure S13. Histograms of probability (percent on logarithmic scale) of finding an H-bonded or halogen cluster with a given number of molecules (a). The calculated averages numbers of molecules in the clusters are displayed in the graphs. Panel (b) shows the distribution of the donor-acceptor HO···O distances of hydrogen bonds in the studied systems.

1.5. Dimer interaction energy

The analysis of intermolecular interactions was carried out on the basis of the structure parameters and interaction energies determined for three dimers: two linear (A and B) and one cyclic (C). Exemplary, optimized structures of 3Cl1P dimers are presented in **Figure S14**.

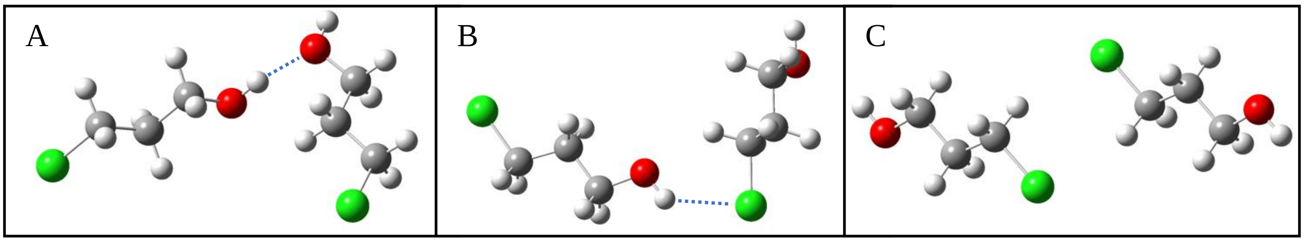

Figure S14. Structure of the optimized dimers of 3Cl1P molecules (B3LYP/6-311G (d,p) calculation model).

Table S5 presents the determined interaction energies between molecules forming dimers, and **Table S6** contains the geometry parameters of the systems. The meaning of the parameters (the distance d and D between the atoms and the angle θ between the bonds) is explained in **Figure S15.**

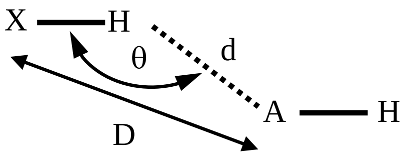

Figure S15. Definition of the geometrical parameters (d, D, θ) of a hydrogen bonded dimer.

The comparison of the determined parameters (**Table S5** – energy, **Table S6** - geometry parameters) with the criteria for classifying hydrogen interactions [6], included in **Table S4**, allows classifying the hydrogen bonds of O-H···O linear dimers of the A-type structure to strong hydrogen bonds (interaction energy E > 5 kcal mol^{-1}), while B dimer with bond between the halogen atom and the hydroxyl group (O-H···X) - to weak hydrogen bonds (E < 4 kcal mol^{-1}). Type C cyclic dimer (E ≈ 2 kcal mol^{-1}) is formed without the participation of hydrogen bonds, and the dominant contribution is made by dipole interactions (antiparallel arrangement of dipoles). Comparing the calculation results of the applied functionals, it can be seen that the correction of long-range interactions contained in the CAM-B3LYP functional significantly affects the estimated interaction energy of molecules forming the

dimers (the energy increases by 0.5 - 1.0 kcal mol^{-1}), but has a much smaller impact on the dimer geometries.

Table S4. Some properties of very strong, strong and weak hydrogen bonds [6]

Parameters	Very strong	Strong	Weak
Bond energy (kcal mol^{-1})	15-40	4-15	< 4
Bond lengths	H—A ≈ X–H	H¨A > X – H	H¨A>>X–H
Lengthening of δ(X–H) [Å]	0.05–0.2	0.01–0.05	<0.01
D(X¨A) range [Å]	2.2–2.5	2.5–3.2	3.0–4.0
d(H¨A) range [Å]	1.2–1.5	1.5–2.2	2.0–3.0
θ(X−H¨A) range (°)	175–180	130–180	90–180

A – acceptor, X-H – donor

Table. S5. Dimer interaction energy in kcal mol^{-1} calculated using B3LYP and CAM-B3LYP electron density functionals with 6-311G(d,p) basis set.

Molecule	B3LYP	CAM-B3LYP
nP		
O...H-O	-5.05	-5.96
3Cl1P		
O...H-O	-5.10	-6.04
Cl…O-H	-2.72	-3.43
Cl…H-C(Cl)	-1.40	-2.03
3Br1P		
O...H-O	-5.11	-6.04
Br…O-H	-2.84	-3.52
Br…H-C(Br)	-1.39	-1.94
3I1P		
O...H-O	-5.14	-6.07
I…O-H	-2.61	-3.24
I…H-C(I)	-1.17	-1.63

Table S6. Dimer geometry parameters calculated using B3LYP and CAM-B3LYP electron density functionals with 6-311G(d,p) basis set.

**O-H$^{\cdots}$O**	B3LYP/6-311G(d.p)				CAM-B3LYP/6-311G(d.p)			
Parameter	nP	3Cl1P	3Br1P	3I1P	nP	3Cl1P	3Br1P	3I1P
O-H [Å]	0.969	0.969	0.969	0.969	0.968	0.968	0.968	0.960
d(H…O) [Å]	1.962	1.955	1.953	1.952	1.915	1.909	1.910	1.910
θ(O-H…O) [º]	175.56	172.45	172.56	172.45	174.67	171.40	171.48	169.39
D(O---O) [Å]	2.929	2.918	2.917	2.916	2.880	2.870	2.870	2.867
vdW criterion $d < r_O+r_H= 2.72$Å	yes	yes	yes	yes	yes	yes	yes	yes
O-H single [Å]	0.961	0.961	0.961	0.961	0.959	0.960	0.960	0.960
δ(O-H) [Å]	0.008	0.008	0.008	0.008	0.009	0.008	0.008	0.008

**O-H$^{\cdots}$X**	B3LYP/6-311G(d,p)			CAM-B3LYP/6-311G(d,p)		
Parameter	3Cl1P	3Br1P	3I1P	3Cl1P	3Br1P	3I1P
O-H [Å]	0.964	0.965	0.964	0.962	0.963	0.963
d(H...X) [Å]	2.636	2.726	3.007	2.591	2.699	2.692
θ(O-H...X) [º]	135.72	138.71	134.05	133.55	135.28	146.03
D(O---X) [Å]	3.393	3.509	3.742	3.328	3.450	3.537
vdW criterion $d < r_X+r_H$	yes $r_{Cl}+r_H$ =2.95	yes $r_{Br}+r_H$=3.05	yes r_I+r_H=3.18	yes $r_{Cl}+r_H$ =2.95	yes $r_{Br}+r_H$=3.05	yes r_I+r_H=3.18
O-H single [Å]	0.961	0.961	0.961	0.960	0.960	0.960
δ(O-H) [Å]	0.003	0.004	0.003	0.002	0.002	0.003

**(X)C-H$^{\cdots}$X**	B3LYP/6-311G(d,p)			CAM-B3LYP/6-311G(d,p)		
Parameter	3Cl1P	3Br1P	3I1P	3Cl1P	3Br1P	3I1P
C-H [Å]	1.089	1.089	1.088	1.088	1.087	1.087
d(H...X) [Å]	3.074	3.183	3.451	3.054	3.130	3.357
θ(C-H...X)	159.11	163.09	170.78	131.59	142.13	165.77
D(C---X) [Å]	4.109	4.236	4.528	3.627	4.0437	4.419
vdW criterion	no	no	no	no	no	no

d < r_C+r_H = 2.90Å						
(X)C-H single [Å]	1.089	1.089	1.088	1.088	1.089	1.087
δ(C-H) [Å]	0.0	0.0	0.0	0.0	0.002	0.0

X – Cl, Br, I; r_H = 1.20, r_C = 1.70, r_O = 1.52, r_{Cl} = 1.75, r_{Br} = 1.85, r_I = 1.98 Å [7]

1.6. Thermal evolution of density and RI

As shown in Figure S16a and b, the temperature dependences of ρ and RI have a linear character for each monoalcohols. Therefore, the experimental data were refined with a linear function, the extrapolation of which allowed to estimate their values at lower temperature range.

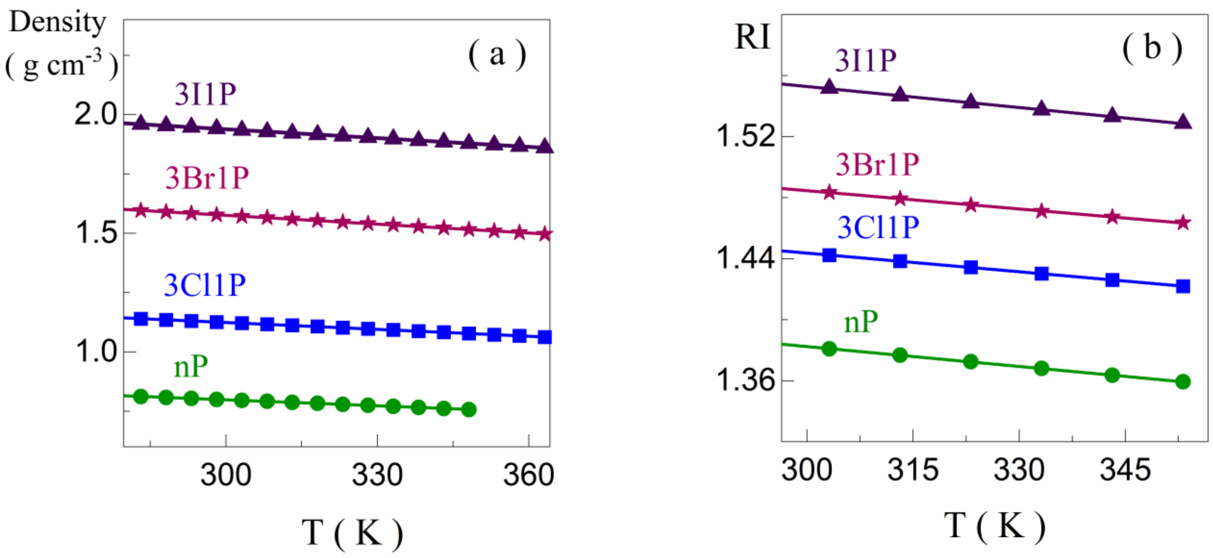

Figure S16. Thermal evolution of density of the studied alcohols (a).Thermal evolution of refractive index of the studied alcohols (b).

1.7. Differential scanning calorimetry

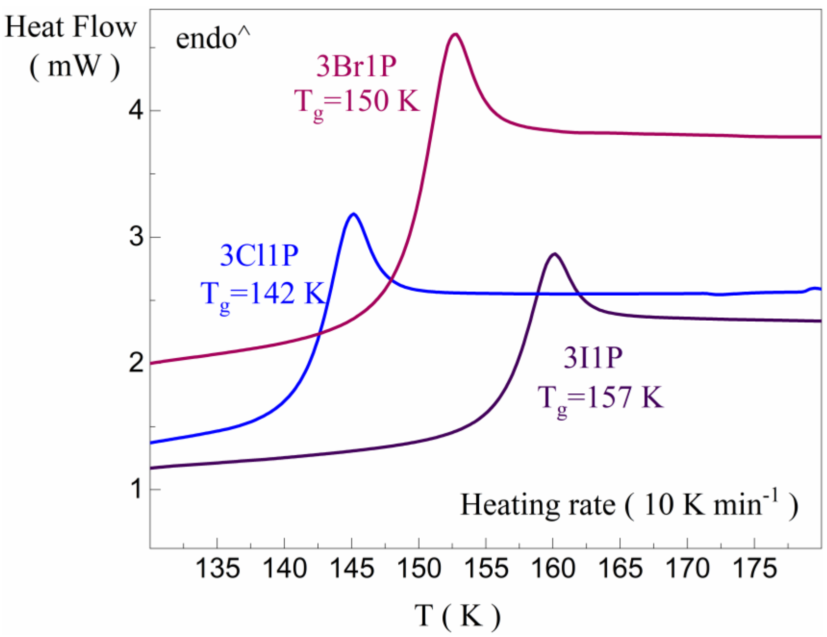

Figure S17. DSC thermograms were collected on heating with a rate of 10 K min^{-1}.

Table S7. Molar Mass (*M*), Glass Transition Temperature (T_g) of the studied alcohols.

Compound	nP	3Cl1P	3Br1P	3I1P
M (g mol^{-1})	60	95	139	186
T_g (K)	98[8]	142	150	157

References

(1) Kao, K. C. Dielectric Phenomena in Solidss. In *Elsevier Academic Press*; London, 2004; pp 92–95.

(2) Havriliak, S.; Negami, S. A Complex Plane Analysis of α-Dispersions in Some Polymer Systems. *J. Polym. Sci. Part C Polym. Symp.* **2007**, *14* (1), 99–117. https://doi.org/10.1002/polc.5070140111.

(3) Davidson, D. W.; Cole, R. H. Dielectric Relaxation in Glycerine. *J. Chem. Phys.* **1950**, *18* (10), 1417–1417. https://doi.org/10.1063/1.1747496.

(4) Gainaru, C.; Meier, R.; Schildmann, S.; Lederle, C.; Hiller, W.; Rössler, E. A.; Böhmer, R. Nuclear-Magnetic-Resonance Measurements Reveal the Origin of the Debye Process in Monohydroxy Alcohols. *Phys. Rev. Lett.* **2010**, *105* (25), 258303. https://doi.org/10.1103/PhysRevLett.105.258303.

(5) Strobl, G. *The Physics of Polymers*; Springer: Berlin, 1997.

(6) Desiraju, G.; Steiner, T. *The Weak Hydrogen Bond*; Oxford University Press: New York, 2001. https://doi.org/10.1093/acprof:oso/9780198509707.001.0001.

(7) Bondi, A. Van Der Waals Volumes and Radii. *J. Phys. Chem.* **1964**, *68* (3), 441–451.

(8) Ramos, M. A.; Kabtoul, B.; Hassaine, M. Calorimetric and Thermodynamic Study of Glass-Forming Monohydroxy Alcohols. *Philos. Mag.* **2011**, *91* (13–15), 1847–1856. https://doi.org/10.1080/14786435.2010.526649.
